# A potential fate decision landscape of the TWEAK/Fn14 axis on stem and progenitor cells: a systematic review

**DOI:** 10.1186/s13287-022-02930-z

**Published:** 2022-06-21

**Authors:** Sijia Wang, Liang Li, Christopher Cook, Yufei Zhang, Yumin Xia, Yale Liu

**Affiliations:** 1 Department of Dermatology, The Second Affiliated Hospital of Xi’an Jiaotong University, 157 Xiwu Road, Xi’an, 710004 Shaanxi China; 2 Department of Thoracic Surgery, The Second Affiliated Hospital of Xi’an Jiaotong University, Xi’an, 710004 Shaanxi China; 3 Division of Immunology and Pathogenesis, Department of Molecular and Cell Biology, University of California, Berkeley, Berkeley, CA USA

**Keywords:** TWEAK/Fn14, Stem and progenitor cells, Fate decision

## Abstract

Stem and progenitor cells (SPCs) possess self-remodeling ability and differentiation potential and are responsible for the regeneration and development of organs and tissue systems. However, the precise mechanisms underlying the regulation of SPC biology remain unclear. Tumor necrosis factor-like weak inducer of apoptosis (TWEAK) acts on miscellaneous cells via binding to fibroblast growth factor-inducible 14 (Fn14) and exerts pleiotropic functions in the regulation of divergent stem cell fates. TWEAK/Fn14 signaling can regulate the proliferation, differentiation, and migration of multiple SPCs as well as tumorigenesis in certain contexts. Although TWEAK’s roles in modulating multiple SPCs are sparsely reported, the systemic effector functions of this multifaceted protein have not been fully elucidated. In this review, we summarized the fate decisions of TWEAK/Fn14 signaling on multiple stem cells and characterized its potential in stem cell therapy.

## Introduction

Tumor necrosis factor (TNF)-like weak inducer of apoptosis (TWEAK or TNFSF12) is a classical member of the TNF ligand superfamily ubiquitously expressed in various tissues and cells. TWEAK exerts miscellaneous biological functions via binding to fibroblast growth factor–inducible 14 (Fn14) to drive cell proliferation, differentiation, migration, and death (apoptosis/necrosis), as well as the stimulation of proinflammatory responses and angiogenesis [1]. Recently, numerous studies have demonstrated that the TWEAK/Fn14 axis plays a crucial role in regulating the cell fates of multiple stems and progenitor cells (SPCs) including pluripotent stem cells like mesenchymal stem cells (MSCs) and embryonic stem cells (ESCs) [2], unipotent stem cells such as liver progenitor cells (LPCs) [3], muscle satellite cells [4], and neural progenitor cells (NSPCs) [5].

SPCs are dedicated undifferentiated cells with self-renewal capacity and are categorized into oligopotent, totipotent, pluripotent, multipotent, and unipotent cells based on their differentiation potential [6]. SPCs can also be assorted into two broad types based on the source of origin: ESCs derived from the inner cell mass of blastocysts, and adult SPCs uncovered in almost every organ [7]. Classically, SPCs possess two properties: the capacity to carry repeated cycles of cell division while preserving their non-differentiated state referred to as self-renewal, and the potential to differentiate into specific cell types, which is referred to as potency [8]. Asymmetric division of SPCs produces one stem cell and one daughter cell that is committed to differentiating into a non-stem cell, a simple but stylish way to maintain equilibrium between self-renewal and differentiation.

SPCs are responsible for the regeneration and development of organs and tissue systems through proliferation and differentiation. The activities of SPCs are regulated by the regional stem cell microenvironment called the “niche”, among which inflammatory cytokines play a critical role in regulating SPC fate [9]. For example, TNF-α induces the proliferation and differentiation of muscle satellite cells [10]. Interleukin (IL)-6 promotes the proliferation of post-natal neural SPCs and is important for their maintenance [11]. The above-mentioned cytokines can also activate signaling pathways, like Janus kinase/signal transducer and activator of transcription (JAK/STAT), Wnt, and Notch to regulate the proliferation and differentiation of SPCs [9, 12]. Though there has been significant progress in unraveling the complexity of stem cell fate decisions, the long-term outcome of stem cell therapy is still unsatisfactory for a series of perplexing fate transforms including immunogenicity, heterogeneity, and tumorigenicity [13]. Thus, a better perception of the functional regulation of common SPCs is still required. Given the pleiotropy of TWEAK, we reviewed the potential fate decisions of multiple SPCs regulated by TWEAK/Fn14 signaling.

## The basic effects of TWEAK/Fn14 interaction

TWEAK, a typical member of the TNF ligand superfamily is initially synthesized as a type II transmembrane protein and then proteolytically cleaved by furin into a soluble variant [14]. TWEAK is constitutively expressed and secreted in multiple tissues and cell types, especially by macrophages and monocytes under inflammatory conditions [15]. Two receptors of TWEAK have been identified so far: the macrophage-derived scavenger receptor CD163 and fibroblast growth factor–inducible 14 (Fn14) [16, 17]. Since CD163 mainly acts as a scavenger receptor [18], Fn14 is the critical receptor mediating TWEAK’s effect. Fn14 is pervasively expressed in various tissues and cell types and highly increased under tissue injury, inflammatory responses, and tissue regeneration [15]. Fn14 contains a singular TNFR-associated factor (TRAF)-binding motif at the cytoplasmic tail, which can attract adaptor proteins TRAFs to initiate canonical downstream signaling cascades such as the nuclear factor-kappaB (NF-κB), mitogen-activated protein kinase (MAPK), phosphatidylinositol 3-kinase (PI3K)-Akt, and Notch pathways to mediate TWEAK signaling [15, 19].

TWEAK/Fn14 signaling can activate both classical and nonclassical NF-κB pathways by recruiting cellular inhibitor of apoptosis proteins (cIAP)-1/2 and TRAFs [4]. Upon TRAFs recruitment, cIAP1/2 is capable of catalyzing the polyubiquitination of receptor-interacting serine/threonine-protein kinase (RIP)-1 to initiate canonical NF-κB signaling [4]. Contrarily, the activation of noncanonical NF-κB, dependent on canonical NF-κB, is attributed to the sequestration of the cIAP1/2-TRAF2 complex to the plasma membrane and leads to the stabilization of cytosolic NF‐κB‐inducing kinase (NIK) [4]. The activated canonical NF-κB pathway can further activate Notch signaling, which is critical in the regulation of muscle satellite cell fate [20]. Further, Notch signaling can maintain pancreatic homeostasis by promoting proliferation and preventing differentiation of pancreatic progenitor cells [21, 22]. The Notch pathway can also promote muscle satellite cell self-renewal by upregulating Hey1 and Hes6 [20] while inhibiting the proliferation and differentiation of muscle satellite cells by repressing the levels of myogenic differentiation antigen (MyoD).

**Fig. 1:**
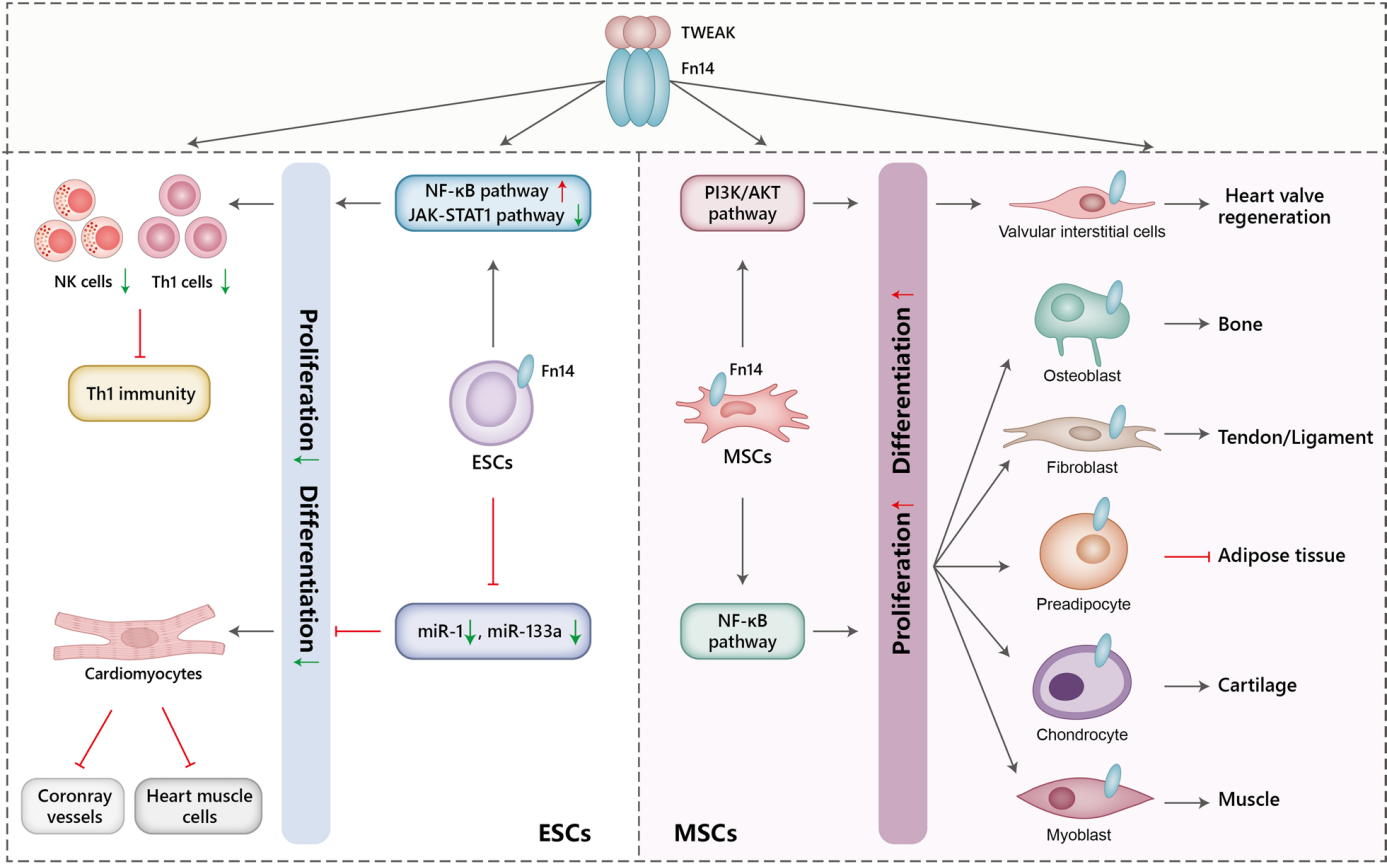
The diagram for TWEAK/Fn14 axis effects on pluripotent stem cells. TWEAK, binding to the Fn14 receptor on embryonic stem cells (ESCs) or mesenchymal stem cells (MSCs), leads to distinct effects on differentiation. TWEAK acts on ESCs to inhibit Th1 immunity, coronary vessel formation, or heart muscle formation via increased NF-κB and decreased JAK-STAT1 signaling. However, TWEAK promotes MSC differentiation into mesenchymal lineage cells via NF-κB or PI3K/Akt. All the lineage cells, including myoblast, chondrocyte, preadipocyte, fibroblast, and osteoblast, express Fn14 and are TWEAK-responsive. TWEAK administration can induce the mesenchymal lineage cells differentiating into corresponding tissues except for preadipocyte. Akt, protein kinase B; ESCs, embryonic stem cells; Fn14, fibroblast growth factor‐inducible 14; JAK, janus kinase; miR, microRNA; MSCs, mesenchymal stem cells; NK, natural killer; NF-κB, nuclear factor kappa-light-chain-enhancer of activated B cells; PI3K, phosphatidylinositol 3-kinase; STAT1, signal transducer and activator of transcription 1; Th1, T helper 1; TWEAK, tumor necrosis factor-like weak inducer of apoptosis

**Fig. 2:**
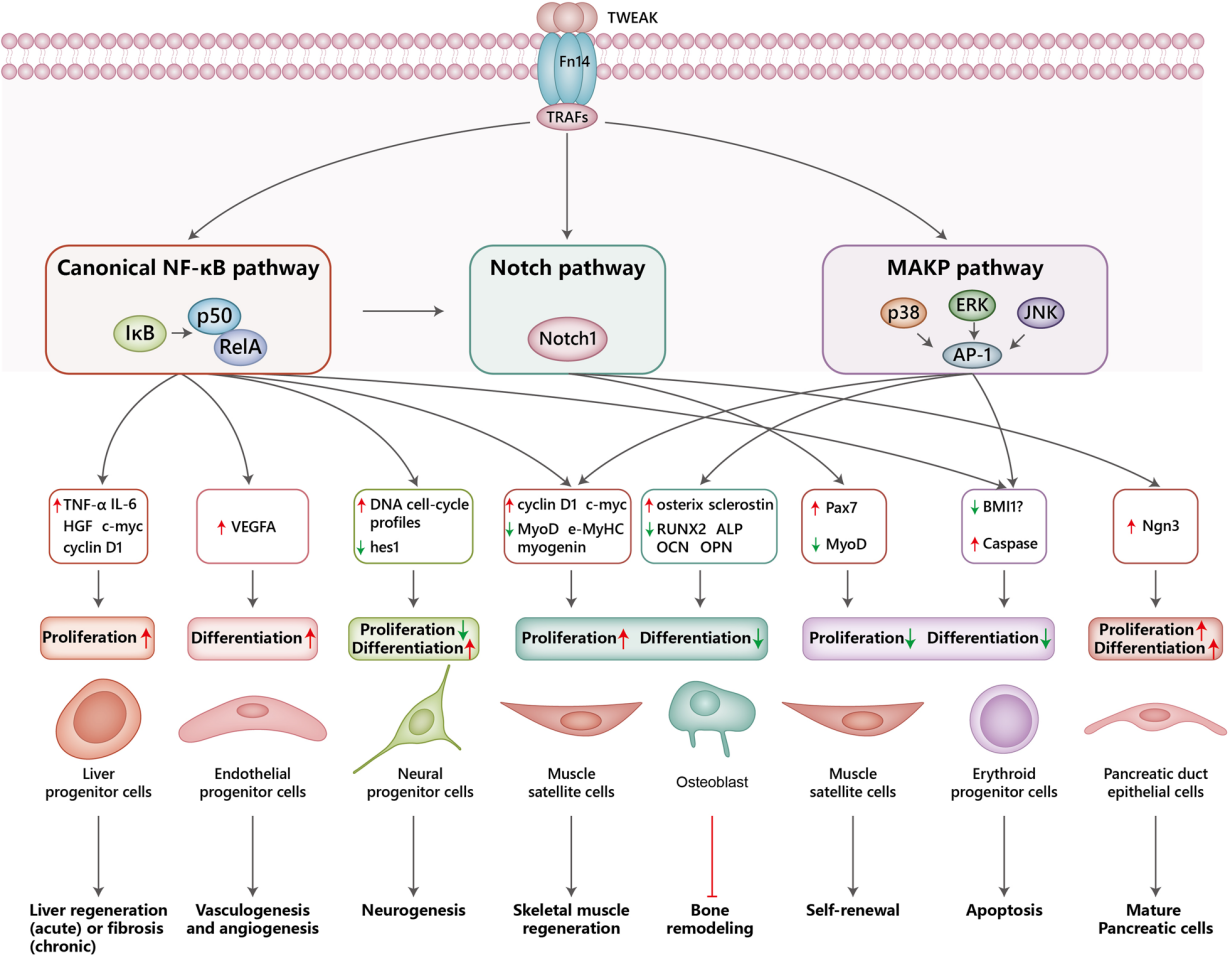
Schematic diagram illustrating effect of the TWEAK/Fn14 axis on multipotent stem/progenitor cells. Binding of trimeric TWEAK to Fn14 receptor leads to the recruitment of cIAPs and various TRAFs resulting in the activation of multiple downstream signaling cascades. TWEAK stimulates the canonical NF-κB pathway which causes the proliferation of liver progenitor cells, differentiation of endothelial progenitor cells, and decreased proliferation and increased differentiation of neural progenitor cells, as well as increased proliferation and decreased differentiation of satellite cells and osteobalsts. TWEAK or TWEAK-activated canonical NF-κB can further activate Notch signaling resulting in decreased proliferation and differentiation of satellite cells but increased proliferation and differentiation of pancreatic duct epithelial cells. TWEAK also activates MAPK signaling cascades resulting in the activation of the AP-1 transcription factor to drive gene expression. The activated genes contribute to increased proliferation and decreased differentiation of satellite cells as well as decreased proliferation and differentiation of erythroid progenitor cells. A20, a NF-κB-dependent gene; AP-1, activator protein-1; Bmi1, BMI1 polycomb ring finger oncogene; cdc2, cell division cycle protein 2; cIAPs, cellular inhibitor of apoptosis proteins; e-MyHC, embryonic myosin heavy chain; ERK, extracellular signal-regulated kinase; Fn14, fibroblast growth factor‐inducible 14; hes1, Hes family BHLH transcription factor 1; IκB, inhibitor of NF-κB; JNK, c-Jun N-terminal kinase; MAPK, mitogen-activated protein kinase; MyoD, myogenic fifferentiation 1; Pax7, paired box 7; NF-κB, nuclear factor kappa-light-chain-enhancer of activated B cells; NGN3, neurogenin 3; NIK, NF‐κB‐inducing kinase; TRAFs, tumor necrosis factor receptor-associated factors; TWEAK, tumor necrosis factor-like weak inducer of apoptosis; VEGFA, vascular endothelial growth factor A

TWEAK can also activate p38 MAPK, extracellular signal-related kinase (ERK)1/2, and Jun N-terminal kinase 1 (JNK1) through several different MAPK kinase kinases (MKKKs). These activated transcription factors act on transcription factor AP-1 to regulate expression of genes involved in TWEAK-regulated proliferation or differentiation including cell-cycle genes (M-Ras, c-myc, cyclin A2, cyclin D1), transcriptional factors (MyoD, Notch), and chemokines (CCL2, CCL5, etc.) [2, 15]. Furthermore, TWEAK can impact cyclins (cyclinD1) and cyclin-dependent kinase (CDK4, CDK6) expression at both the protein and mRNA level and decrease the expression of cyclin-dependent kinase inhibitors through PI3K-Akt signaling pathways, and thus significantly regulate proliferation. In sum, TWEAK/Fn14 signaling can modulate the fate decision of multiple SPCs by activating downstream signaling pathways and regulating the proliferation and differentiation of SPCs (Figs. 1 and 2).

## The fate decisions of TWEAK/Fn14 signaling on pluripotent SCs

Pluripotent SCs, including ESCs and MSCs, have the ability to undergo self-renewal and give rise to all cells in the human body. Therefore, they are attractive for their potential preclinical and clinical therapeutic application to treat a wide array of diseases. Current research has found TWEAK can regulate the ESCs and MSCs proliferation or differentiation, therefore regulating the ESC-associated Th1 immune response or myogenesis as well as mesenchymal lineage differentiation [2, 23–25].

### TWEAK inhibits ESC differentiation into Th1 immunity or muscle

Fn14 is expressed by ESCs [26] and ESC-edited with TWEAK deletion vectors mice showed increased natural killer (NK) cells in secondary lymphoid organs and hypersensitivity to systemic lipopolysaccharide (LPS) challenge because of redundant interferon (IFN)-γ and IL-12 production [27]. The effects are mediated by the induced NF-κB and ablated JAK/STAT-1 signaling and are reversed by TWEAK stimulation. ESC-edited TWEAK^−/−^ mice also developed expanded memory and T helper 1 (Th1) cells upon aging and B16 melanoma cells challenge [27]. Therefore, TWEAK can act on ESCs to inhibit the activation of Th1 immunity by repressing NK cells. TWEAK can also inhibit the expression of miR-1 and miR-133a in ESCs, which are critical to stimulate myoblast proliferation and promote myoblast and cardiomyocyte differentiation into coronary vessels and heart muscle cells, to preventing skeletal muscle, heart vessel, and muscle regeneration [23, 28–30]. Generally, TWEAK can act on ESCs to regulate Th1 adaptive immunity or inhibit ESC differentiation into heart and skeletal muscle, while targeting TWEAK can strengthen Th1 immune response and myogenesis (Fig. 1).

### TWEAK promotes both proliferation and differentiation of MSCs

The effects of the TWEAK/Fn14 signaling pathway on MSCs were also investigated. TWEAK promotes MSC proliferation by upregulating the expression of pro-survival genes such as *A20* and *cIAP2*, pro-proliferation genes such as *cdc2*, *cyclin* A2, *survivin*, and *MAD2*, and adhesion genes such as *ICAM-1* and *VCAM-1n* [2]. Proliferated MSCs can differentiate into myoblasts, chondrocytes, preadipocytes, fibroblasts, and osteoblasts via the NF-κB pathway [2]. TWEAK can also bind to all progenitor cells of the mesenchymal lineage and differentiate into the corresponding tissue cells except for preadipocytes [2, 24]. Additionally, TWEAK promotes MSC differentiation into valvular interstitial cells (VICs) depending on the activation of the PI3K/Akt signaling pathway [25]. The VICs can increase smooth muscle actin (SMA) expression, promote the co-alignment of α-actinin, drive stress fiber (F-actin) into bundles, and increase extracellular matrix protein synthesis [25]. VICs are the predominant cell population in mature valves and are responsible for the maintenance of normal valve integrity, making them a promising seeding cell of tissue-engineered heart valves. These effects of the TWEAK/Fn14 axis on MSC proliferation and differentiation may provide an innovative therapeutic strategy for the treatment of mesenchymal-associated diseases (Fig. 1).

## The fate decisions of TWEAK/Fn14 signaling on unipotent SPCs

### TWEAK promotes LPC proliferation to induce chronic liver damage

LPCs are elicited under reduced functional liver mass or impaired hepatocyte replication, and go through the process of proliferating, migrating into the parenchyma, and differentiating into either hepatocytes or biliary epithelial cells to repair the damage [31]. This process is intricately regulated and has been reported to involve several factors, including TNF-α, interleukin-6 (IL-6), and TWEAK; however, only TWEAK has been found to join the ranks of key LPC mediators [32]. TWEAK-administrated liver progenitor cell line (603B cells) have increased expression of Fn14 protein as well as hepatocytic progenitor markers, like alpha-fetoprotein (AFP) and leucine rich repeat containing G protein-coupled receptor 5 (LGR5) [32]. TWEAK^−/−^ mice, mice treated with an inhibitory anti-TWEAK antibody, or Fn14^−/−^ mice show reduced damage-induced proliferation of LPCs [3]. These effects are attributed to TWEAK-induced expression of TNF-α, IL-6, and hepatocyte growth factor (HGF) as well as cell cycle proteins (cyclin D1 and c-myc), all of which are regulated by the NF-κB pathway [33]. These results suggest TWEAK is a promising target for liver repair in the acute stage (Fig. 2).

Besides liver repair, the number of LPCs was also directly associated with the severity of fibrosis [34]. Prolonged TWEAK/Fn14 activation selectively exerts a mitogenic effect on LPCs and can drive chronic liver damage such as chronic hepatitis, nonalcoholic steatohepatitis, and alcoholic liver disease [3, 34]. TWEAK promotes the proliferation of LPCs by binding to Fn14 which then activates NF-κB driving the upregulation and production of numerous cell cycle regulatory factors, like cyclin D1 and c-myc [3, 34]. TWEAK stimulates the proliferation of LPC populations both in overexpressed transgenic mice and transiently expressed normal adult mice [3], while Fn14^−/−^ mice or mice administered an anti-TWEAK mAb show attenuated LPC expansion and liver injury [3]. The same effects are observed in a murine choline-deficient, ethionine-supplemented (CDE) model of chronic liver injury after TWEAK stimulation [34]. A carbon tetrachloride–motivated fibrosis study showed that remedial TWEAK inhibition diminishes LPC expansion, leading to a strongly downregulated fibrogenic response and augmenting fibrotic liver regeneration [35]. Overall, current data strongly suggest that the TWEAK/Fn14 effects on LPCs are protective in acute liver damage by promoting liver repair but pathogenetic in chronic liver damage via the induction of liver fibrosis [3, 34, 35], indicating the double-edged roles of TWEAK/Fn14 in liver diseases (Fig. 2).

### TWEAK induces Endothelial progenitor cell (EPC) differentiation

EPCs are capable of differentiation into mature endothelial cells (ECs) to support vascular endothelial repair and angiogenesis [36]. Therefore, EPCs have been postulated as valuable cellular candidates or therapeutic targets to improve cardiovascular disease, such as acute myocardial infarction (AMI). TWEAK is significantly escalated in patients and mice with AMI compared to healthy controls [37, 38]. Transplantation of TWEAK-pretreated EPCs into AMI mice significantly improved cardiac function, alleviated AMI, and facilitated the differentiation of EPCs to form vessels [38]. Thus, TWEAK can protect the heart from ischemic damage [36]. Fn14 or NF-κB pathway blockage decreased the protective effect of EPCs on AMI [38]. Furthermore, TWEAK promotes EPC viability, migration to injured tissue, tube formation, and vascular endothelial growth factor A (VEGFA) generation in vivo or in vitro via the Fn14-NF-κB pathway [38] (Fig. 2). These findings indicate that the activation of the TWEAK-Fn14-NF-κB signaling pathway induces EPC differentiation and vasculogenesis, which are beneficial for the treatment of AMI.

The effects of TWEAK on EPCs can also promote wound healing. TWEAK promotes EPC migration into the wound area and accelerates the healing process by driving the differentiation of EPCs into mature endothelial cells [38, 39], which are helpful for vasculogenesis and angiogenesis. Infiltrating macrophages in the wound area can further produce TWEAK to act on EPCs, forming a virtuous circle in wound healing [40].

### TWEAK promotes the differentiation of neural progenitor cells (NPCs) for neurogenesis

NPCs play a pivotal role in both the neurodevelopment and repair of nervous tissue upon injury and may be the best choice for the treatment of spinal cord injury [41]. Fn14 is expressed by NPCs and the TWEAK/Fn14 pathway can distinctly regulate NPCs depending on their developmental stage [5, 42]. TWEAK-induced neurite elongation did not alter proliferation or differentiation on embryonic day 12 (E12) NPCs. Yet while TWEAK does not affect neurite outgrowth, it decreases proliferation in postnatal day 1 (PN1) NPCs by regulating DNA cell-cycle profiles [42]. TWEAK also inhibited the proliferation of adult NPCs yet induced their differentiation by downregulating helix-loop-helix gene *hes1* via the NF-κB signaling pathway [5]. Fn14 blockage reduced neurogenesis in adult Fn14-deficient mice. These studies demonstrate the pivotal role of the TWEAK/Fn14 axis in regulating NPCs and promoting neurogenesis (Fig. 2). Future studies examining the mechanism underlying developmental changes in adult NPCs responsive to TWEAK signaling may provide clues to the stem cell therapy of degenerative nerve diseases or nerve damage.

### TWEAK promotes proliferation but inhibits differentiation

#### Muscle satellite cells

Muscle satellite cells are adult muscle precursor cells located under the basal lamina of the muscle fiber in a mitotically quiescent state [43]. These cells are responsible for both muscle formation during development and repair as well as regeneration of adult myofibers in response to injury and muscular adaptation to exercise [44, 45]. Once activated, the muscle satellite cells undergo expansion as well as progression to become fusion-competent myoblasts, which undergo some round of proliferation and then exit the cell cycle to enter a highly orchestrated differentiation program and finally fuse into new multinucleated muscle fibers [46].

Current research found muscle satellite cells can express Fn14 and are TWEAK responsive. The TWEAK and Fn14 levels are very low in quiescent muscle tissue but are dramatically upregulated following muscle damage [47]. The TWEAK/Fn14 pathway governs muscle satellite cells’ decisions to self-renew, proliferate, differentiate, and regulate muscle regeneration. TWEAK shows indiscriminate and extremely state-dependent effects on muscle regeneration and homeostasis verified by in vivo and in vitro experiments of muscle progenitor cells [2, 20, 47–50]. At sufficiently high concentration (≥ 100 ng/mL), TWEAK promotes the proliferation of C2C12 cells while inhibiting differentiation, which can be reversed by generating Fn14-deficient myoblasts [2, 48]. Further, TWEAK activates the canonical NF-κB pathway via binding to Fn14 in myoblasts and promotes the proliferation of muscle satellite cells by upregulating cell cycle-related genes such as cyclin D1 and c-myc, while inhibiting myogenesis by decreasing the levels of MyoD and myogenin. TWEAK treatment in myoblasts also drives the transcription factors activator protein-1 (AP-1), p44-p42 MAPK, and Notch, which upregulate proliferation genes but downregulate differentiation pathways [22, 49]. Silencing of Fn14 expression in C2C12 myoblasts [49] or deleting Fn14 in mice impaired muscle satellite cell-driven skeletal muscle regeneration [2]. On the other hand, low concentrations of TWEAK (10 ng/mL) robustly promoted myoblast fusion without affecting their proliferation or differentiation via the preferential induction of noncanonical NF-κB signaling [50]. TWEAK can also inhibit the proliferation and differentiation of muscle satellite cells through Notch signaling. Muscle satellite cell-specific NICD^OE^ mice studies found that Notch activation inhibits myoblast proliferation via upregulating Pax7 [51], which can stop MyoD function from inducing the differentiation of muscle satellite cells. These results suggest that TWEAK activated canonical NF-κB, AP-1, and Notch pathways to induce C2C12 cell to proliferate. The ability of the TWEAK/Fn14 pathway to broadly regulate these critical properties of muscle progenitor cells together with muscle regeneration might therefore present an opportunity for “TWEAKing" existing approaches for cell-based regenerative therapies, particularly in various muscle tissue injury and disease settings (Fig. 2).

#### Osteoblasts

Osteoblasts, one of the mesenchymal lineage progenitors, are involved in the bone remodeling process and regeneration following injury. Human osteoblasts express Fn14 and TWEAK exposure enhances osteoblast proliferation via the upregulation of osterix and the downregulation of runt-related transcription factor-2 (RUNX2) levels [52]. Contrarily, TWEAK suppressed the expression of differentiation genes, such as osteocalcin (OCN), alkaline phosphatase (ALP), and osteopontin (OPN) via the MAPK extracellular signal-regulated kinase (ERK) pathway in MC3T3-E1 cells [52]. Because of the role of RUNX2 in controlling osteoblast proliferation and osterix in promoting cell proliferation and decreasing expression of OCN and ALP, the effects of TWEAK on osteoblast proliferation and differentiation may be explained partially by the reciprocal regulation of RUNX2 and osterix. TWEAK can induce the expression of sclerostin, a bone differentiation inhibitor, in osteoblasts through the JNK and ERK1/2 pathways in vitro or ex vivo [52, 53] (Fig. 2). TWEAK/Fn14 interplay also inhibits BMP-2-induced differentiation, which is reversed by Fn14-Fc chimera and MAPK inhibitor PD98059 [54]. Generally, TWEAK induces the proliferation and inhibits the differentiation of osteoblasts to impede osteoblastogenesis [52, 54], indicating that inhibiting TWEAK may be an innovative therapeutic target for bone-related diseases.

### TWEAK promotes both proliferation and differentiation of pancreatic duct epithelial cells

The pancreatic ductal epithelial cells serve as progenitor cells and can invoke insulin-producing β-cells in rodents [55, 56]. Fn14 is expressed at relatively low levels in adult healthy pancreatic ducts but upregulated after injury, resulting in the proliferation of duct epithelial cell progenitors followed by differentiation to mature pancreatic cell types, including β-cells [57]. Fc-TWEAK administration can promote proliferation of pancreatic ductal epithelial cells in normal adult mouse pancreas through its receptor Fn14, while Fn14 KO mice attenuated this proliferation [57]. Short-term TWEAK treatment induces transient neurogenin 3 (NGN3) expression in the healthy adult mouse pancreas without islet hormone expression, but long-term TWEAK injection results in some hormone-expressing cells (insulin^+^ or glucagon^+^) within the duct epithelium. Serum TWEAK level also decreased in type 2 diabetes (T2D) patients [58]. These results suggest persisting TWEAK can promote duct epithelial cell progenitors to differentiate into mature pancreatic cell types, including β-cells, and that the TWEAK-Fn14 interaction could potentially facilitate the production of new endocrine beta cells and provide clues for treating T2D [57] (Fig. 2).

### TWEAK inhibits proliferation and differentiation of erythroid progenitors

TWEAK and Fn14 are expressed by erythroid progenitors, and exposure to recombinant TWEAK can inhibit erythroblast proliferation and differentiation and lead to apoptosis [59, 60]. BMI1 polycomb ring finger oncogene (Bmi1) is critical for the survival of erythroid cells while Bmi1 deficiency results in the apoptosis of erythroid progenitor cells [61]. Given p38 MAPK downregulates BMI1 [62], we speculated that TWEAK promotes erythroid progenitor cell apoptosis via p38 MAPK. IFN-γ can inhibit the growth and differentiation of erythroid precursor cells and mediates hematopoietic suppression, which can be partially reverted with TWEAK blockage [59, 60]. TWEAK-triggered NF-κB can induce caspase expression while caspase signaling mediates the effects of IFN-γ, which forms a circuit to inhibit hematopoiesis [63]. The reduction of red blood cells in patients with systemic lupus erythematosus or in the spontaneous mouse model of lupus (NZB mice), and the increased red blood cells in atherosclerotic unstable plaque along with increased TWEAK may further support the roles of TWEAK in anemia [64–67]. All in all, TWEAK can inhibit proliferation and differentiation and promote the apoptosis of erythroid progenitors, while targeting TWEAK may help to treat anemia (Fig. 2).

## The TWEAK/Fn14 axis in tumorigenesis

The roles of the TWEAK/Fn14 axis in SPC fate decisions have elicited the concern of tumorigenesis. It is authentic that certain tumors contain a small population of so-called tumor or cancer stem cells that can self-renew and drive tumorigenesis. TWEAK/Fn14 signaling can lead to potential tumorigenesis by regulating cancer stem cells, verified by some neoplastic and preneoplastic disorders. TWEAK-Fn14 interaction can activate NF-κB or induce matrix metalloprotease 9 (MMP-9) to aggravate tumorigenesis in neuroblastoma [68]. TWEAK can potentially regulate the self-renewal and differentiation of the glioma stem cell (GSC) in glioblastoma and induce the resistance of these tumors to conventional therapies as well as recurrent disease [69]. The TWEAK/Fn14 pathway can also affect the cholangiocarcinoma niche by recruiting and phenotyping macrophages as well as proliferating cancer-associated fibroblasts [70]. However, TWEAK/Fn14 can repress the development of squamous cervical carcinoma even though there are cancer stem cells [71, 72]. TWEAK/Fn14 is also thought to have a protective role in regulating acute intestinal inflammation and preventing colitis-associated tumorigenesis in the setting of inflammatory bowel disease (IBD) [73]. It has been known that upon long-term inflammation or insults in a niche environment, the SCs may mutate and transform to become the origin of the malignancy [74]. This, in concert with the aforementioned findings, indicates that the tumorigenesis during the process of stem cell proliferation and differentiation induced by the TWEAK/Fn14 signal is probably dependent on cell context and whether a given DNA mutation exists in the stem cells.

## Conclusions

Increased clinical trials for stem cell-based therapies make it an emerging research topic to investigate the SPC fate decisions. Therefore, it is an intriguing and timely topic to clarify the interaction between the TWEAK/Fn14 axis and SPC fate decisions. Studies implemented using knockout mice or TWEAK/Fn14 antagonists verified the regulation of TWEAK/Fn14 signaling on SPC proliferation and differentiation. Further, accumulating evidence advocates for the therapeutic potential of TWEAK/Fn14 in tissue damage. However, in terms of targeting TWEAK/Fn14 in stem cell therapy, the different effects of TWEAK/Fn14 on pluripotent and unipotent stem cells should be considered, even though most proliferation or differentiation is regulated by the TWEAK-activated NF-κB pathway. It should also be considered that tumorigenesis may be induced, although the outcome is cell context-dependent. Moreover, the precise effect of TWEAK on several SPCs both in vitro and in vivo has not been completely elucidated, such as epidermal stem cells. Finally, our investigation of TWEAK/Fn14 biology in SPCs is mainly based on data from conventional cell culture and in vivo model systems. Further studies are necessary to unravel the multifaceted and complex roles of the TWEAK-Fn14 axis in order to transition from bench to bedside. Overall, TWEAK/Fn14 signaling modulates the proliferation and differentiation of SPCs by the activation of multiple cytokines and downstream signals, which are crucial components in the SPC “niche” as well as SPC fates.

## Data Availability

Not applicable.

## Abbreviations

AFP: Alpha-fetoprotein
ALP: Alkaline phosphatase
AMI: Acute myocardial infarction
CCL: C–C motif ligand
cIAP: Cellular inhibitor of apoptosis proteins
ERK: Extracellular signal-related kinase
EPCs: Endothelial progenitor cells
ESCs: Embryonic stem cells
Fn14: Fibroblast growth factor-inducible 14
GSC: Glioma stem cell
LGR5: Leucine rich repeat containing G protein-coupled receptor 5
IL-6: Interleukin-6
LPS: Lipopolysaccharide
JAK/STAT1: Janus kinase/signal transducer and activator of transcription1
JNK: Jun N-terminal kinase
LPCs: Liver progenitor cells
MAPK: Mitogen-activated protein kinase
miR: MicroRNA
MKKKs: MAPK kinase kinases
MSCs: Mesenchymal stem cells
MyoD: Myogenic differentiation antigen
NF-κB: Nuclear factor-kappaB
NK: Natural killer
OCN: Osteocalcin
PI3K: Phosphatidylinositol 3-kinase
RUNX2: Runt-related transcription factor-2
SPCs: Stem and progenitor cells
SMA: Smooth muscle actin
Th1: T helper 1
TNF-α: Tumor necrosis factor-α
TRAF: TNFR-associated factor
TWEAK: Tumor necrosis factor-like weak inducer of apoptosis
VEGFA: Vascular endothelial growth factor A
VICs: Valvular interstitial cells

## References

[1] Burkly LC, Michaelson JS, Zheng TS (2011). TWEAK/Fn14 pathway: an immunological switch for shaping tissue responses. Immunol Rev.

[2] Girgenrath M, Weng S, Kostek CA, Browning B, Wang M, Brown SAN (2006). TWEAK, via its receptor Fn14, is a novel regulator of mesenchymal progenitor cells and skeletal muscle regeneration. EMBO J.

[3] Jakubowski A, Ambrose C, Parr M, Lincecum JM, Wang MZ, Zheng TS (2005). TWEAK induces liver progenitor cell proliferation. J Clin Invest.

[4] Enwere EK, Lacasse EC, Adam NJ, Korneluk RG (2014). Role of the TWEAK-Fn14-cIAP1-NF-κB signaling axis in the regulation of myogenesis and muscle homeostasis. Front Immunol.

[5] Schölzke MN, Röttinger A, Murikinati S, Gehrig N, Leib C, Schwaninger M (2011). TWEAK regulates proliferation and differentiation of adult neural progenitor cells. Mol Cell Neurosci.

[6] Al-Suhaimi EA, Al-Khater K (2019). Functions of stem cells of thyroid glands in health and disease. Rev Endocr Metab Disord.

[7] Jin J (2017). Stem cell treatments. JAMA.

[8] López-Lázaro M (2018). The stem cell division theory of cancer. Crit Rev Oncol Hematol.

[9] Naik S, Larsen SB, Cowley CJ, Fuchs E (2018). Two to Tango: Dialog between immunity and stem cells in health and disease. Cell.

[10] Alvarez AM, DeOcesano-Pereira C, Teixeira C, Moreira V (2020). IL-1β and TNF-α modulation of proliferated and committed myoblasts: IL-6 and COX-2-derived prostaglandins as key actors in the mechanisms involved. Cells.

[11] Storer MA, Gallagher D, Fatt MP, Simonetta JV, Kaplan DR, Miller FD (2018). Interleukin-6 regulates adult neural stem cell numbers during normal and abnormal post-natal development. Stem Cell Rep.

[12] Nusse R, Clevers H (2017). Wnt/β-catenin signaling, disease, and emerging therapeutic modalities. Cell.

[13] Yamanaka S (2020). Pluripotent stem cell-based ccell therapy-promise and cchallenges. Cell Stem Cell.

[14] Trebing J, Arana JAC, Salzmann S, Wajant H (2014). Analyzing the signaling capabilities of soluble and membrane TWEAK. Methods Mol Biol.

[15] Wang X, Xiao S, Xia Y (2017). Tumor necrosis factor receptor mediates fibroblast growth factor-inducible 14 ssignaling. Cell Physiol Biochem.

[16] Bover LC, Cardó-Vila M, Kuniyasu A, Sun J, Rangel R, Takeya M (2007). A previously unrecognized protein-protein interaction between TWEAK and CD163: potential biological implications. J Immunol.

[17] Wiley SR, Cassiano L, Lofton T, Davis-Smith T, Winkles JA, Lindner V (2001). A novel TNF receptor family member binds TWEAK and is implicated in angiogenesis. Immunity.

[18] Van Gorp H, Delputte PL, Nauwynck HJ (2010). Scavenger receptor CD163, a Jack-of-all-trades and potential target for cell-directed therapy. Mol Immunol.

[19] Zhang Y, Zeng W, Xia Y (2021). TWEAK/Fn14 axis is an important player in fibrosis. J Cell Physiol.

[20] Ogura Y, Mishra V, Hindi SM, Kuang S, Kumar A (2013). Proinflammatory cytokine tumor necrosis factor (TNF)-like weak inducer of apoptosis (TWEAK) suppresses satellite cell self-renewal through inversely modulating Notch and NF-κB signaling pathways. J Biol Chem.

[21] Ahnfelt-Rønne J, Hald J, Bødker A, Yassin H, Serup P, Hecksher-Sørensen J (2007). Preservation of proliferating pancreatic progenitor cells by Delta-Notch signaling in the embryonic chicken pancreas. BMC Dev Biol.

[22] Akahori H, Karmali V, Polavarapu R, Lyle AN, Weiss D, Shin E (2015). CD163 interacts with TWEAK to regulate tissue regeneration after ischaemic injury. Nat Commun.

[23] Panguluri SK, Bhatnagar S, Kumar A, McCarthy JJ, Srivastava AK, Cooper NG (2010). Genomic profiling of messenger RNAs and microRNAs reveals potential mechanisms of TWEAK-induced skeletal muscle wasting in mice. PLoS ONE.

[24] Tiller G, Fischer-Posovszky P, Laumen H, Finck A, Skurk T, Keuper M (2009). Effects of TWEAK (TNF superfamily member 12) on differentiation, metabolism, and secretory function of human primary preadipocytes and adipocytes. Endocrinology.

[25] Huang W, Xiao D-Z, Wang Y, Shan Z-X, Liu X-Y, Lin Q-X (2014). Fn14 promotes differentiation of human mesenchymal stem cells into heart valvular interstitial cells by phenotypic characterization. J Cell Physiol.

[26] Ramalho-Santos M, Yoon S, Matsuzaki Y, Mulligan RC, Melton DA (2002). “Stemness”: transcriptional profiling of embryonic and adult stem cells. Science.

[27] Maecker H, Varfolomeev E, Kischkel F, Lawrence D, LeBlanc H, Lee W (2005). TWEAK attenuates the transition from innate to adaptive immunity. Cell.

[28] van Rooij E, Liu N, Olson EN (2008). MicroRNAs flex their muscles. Trends Genet.

[29] Chen J-F, Mandel EM, Thomson JM, Wu Q, Callis TE, Hammond SM (2006). The role of microRNA-1 and microRNA-133 in skeletal muscle proliferation and differentiation. Nat Genet.

[30] Kura B, Kalocayova B, Devaux Y, Bartekova M (2020). Potential clinical implications of miR-1 and miR-21 in heart disease and cardioprotection. Int J Mol Sci.

[31] Lombard CA, Prigent J, Sokal EM (2013). Human liver progenitor cells for liver repair. Cell Med.

[32] Karaca G, Swiderska-Syn M, Xie G, Syn W-K, Krüger L, Machado MV (2014). TWEAK/Fn14 signaling is required for liver regeneration after partial hepatectomy in mice. PLoS One.

[33] Yin J, Lee JH, Zhang J, Gao Z, Polotsky VY, Ye J (2014). Regulation of hepatocyte growth factor expression by NF-κB and PPARγ in adipose tissue. Am J Physiol Endocrinol Metab.

[34] Tirnitz-Parker JEE, Viebahn CS, Jakubowski A, Klopcic BRS, Olynyk JK, Yeoh GCT (2010). Tumor necrosis factor-like weak inducer of apoptosis is a mitogen for liver progenitor cells. Hepatology.

[35] Tirnitz-Parker JEE, Olynyk JK, Ramm GA (2014). Role of TWEAK in coregulating liver progenitor cell and fibrogenic responses. Hepatology.

[36] Chong MSK, Ng WK, Chan JKY (2016). Concise Review: endothelial progenitor cells in regenerative medicine: applications and challenges. Stem Cells Transl Med.

[37] Chorianopoulos E, Jarr K, Steen H, Giannitsis E, Frey N, Katus HA (2010). Soluble TWEAK is markedly upregulated in patients with ST-elevation myocardial infarction and related to an adverse short-term outcome. Atherosclerosis.

[38] Sheng Z, Ju C, Li B, Chen Z, Pan X, Yan G (2018). TWEAK promotes endothelial progenitor cell vasculogenesis to alleviate acute myocardial infarction via the Fn14-NF-κB signaling pathway. Exp Ther Med.

[39] Kaushik K, Das A (2019). Endothelial progenitor cell therapy for chronic wound tissue regeneration. Cytotherapy.

[40] Liu J, Liu Y, Peng L, Li J, Wu K, Xia L (2019). TWEAK/Fn14 signals mediate burn wound repair. J Invest Dermatol.

[41] Yousefifard M, Rahimi-Movaghar V, Nasirinezhad F, Baikpour M, Safari S, Saadat S (2016). Neural stem/progenitor cell transplantation for spinal cord injury treatment; A systematic review and meta-analysis. Neuroscience.

[42] Hamill CA, Michaelson JS, Hahm K, Burkly LC, Kessler JA (2007). Age-dependent effects of TWEAK/Fn14 receptor activation on neural progenitor cells. J Neurosci Res.

[43] Laumonier T, Menetrey J (2016). Muscle injuries and strategies for improving their repair. J Exp Orthop..

[44] Chen X, Xue G, Zhao J, Zhang Y, Zhang S, Wang W, Li Y, Yuan J, He L, Chan CY, Liu Y, Chen W, Zhao Y, Hu P, Sun H, Kwok CK, Wang H (2022). Lockd promotes myoblast proliferation and muscle regeneration via binding with DHX36 to facilitate 5' UTR rG4 unwinding and Anp32e translation. Cell Rep..

[45] Boyer JG, Huo J, Han S, Havens JR, Prasad V, Lin BL, Kass DA, Song T, Sadayappan S, Khairallah RJ, Ward CW, Molkentin JD (2022). Depletion of skeletal muscle satellite cells attenuates pathology in muscular dystrophy. Nat Commun..

[46] Relaix F, Zammit PS (2012). Satellite cells are essential for skeletal muscle regeneration: the cell on the edge returns centre stage. Development.

[47] Mittal A, Bhatnagar S, Kumar A, Paul PK, Kuang S, Kumar A (2010). Genetic ablation of TWEAK augments regeneration and post-injury growth of skeletal muscle in mice. Am J Pathol.

[48] Dogra C, Changotra H, Mohan S, Kumar A (2006). Tumor necrosis factor-like weak inducer of apoptosis inhibits skeletal myogenesis through sustained activation of nuclear factor-kappaB and degradation of MyoD protein. J Biol Chem.

[49] Dogra C, Hall SL, Wedhas N, Linkhart TA, Kumar A (2007). Fibroblast growth factor inducible 14 (Fn14) is required for the expression of myogenic regulatory factors and differentiation of myoblasts into myotubes. Evidence for TWEAK-independent functions of Fn14 during myogenesis. J Biol Chem.

[50] Enwere EK, Holbrook J, Lejmi-Mrad R, Vineham J, Timusk K, Sivaraj B (2012). TWEAK and cIAP1 regulate myoblast fusion through the noncanonical NF-κB signaling pathway. Sci Signal.

[51] Wen Y, Bi P, Liu W, Asakura A, Keller C, Kuang S (2012). Constitutive Notch activation upregulates Pax7 and promotes the self-renewal of skeletal muscle satellite cells. Mol Cell Biol.

[52] Vincent C, Findlay DM, Welldon KJ, Wijenayaka AR, Zheng TS, Haynes DR (2009). Pro-inflammatory cytokines TNF-related weak inducer of apoptosis (TWEAK) and TNFalpha induce the mitogen-activated protein kinase (MAPK)-dependent expression of sclerostin in human osteoblasts. J Bone Miner Res.

[53] Suen PK, Qin L (2016). Sclerostin, an emerging therapeutic target for treating osteoporosis and osteoporotic fracture: A general review. J Orthop Translat.

[54] Ando T, Ichikawa J, Wako M, Hatsushika K, Watanabe Y, Sakuma M (2006). TWEAK/Fn14 interaction regulates RANTES production, BMP-2-induced differentiation, and RANKL expression in mouse osteoblastic MC3T3-E1 cells. Arthritis Res Ther.

[55] Bonner-Weir S, Toschi E, Inada A, Reitz P, Fonseca SY, Aye T (2004). The pancreatic ductal epithelium serves as a potential pool of progenitor cells. Pediatr Diabetes.

[56] Li W-C, Rukstalis JM, Nishimura W, Tchipashvili V, Habener JF, Sharma A (2010). Activation of pancreatic-duct-derived progenitor cells during pancreas regeneration in adult rats. J Cell Sci.

[57] Wu F, Guo L, Jakubowski A, Su L, Li W-C, Bonner-Weir S (2013). TNF-like weak inducer of apoptosis (TWEAK) promotes beta cell neogenesis from pancreatic ductal epithelium in adult mice. PLoS One.

[58] Díaz-López A, Chacón MR, Bulló M, Maymó-Masip E, Martínez-González MA, Estruch R (2013). Serum sTWEAK concentrations and risk of developing type 2 diabetes in a high cardiovascular risk population: a nested case-control study. J Clin Endocrinol Metab.

[59] Felli N, Pedini F, Zeuner A, Petrucci E, Testa U, Conticello C (2005). Multiple members of the TNF superfamily contribute to IFN-gamma-mediated inhibition of erythropoiesis. J Immunol.

[60] Libregts SF, Gutiérrez L, de Bruin AM, Wensveen FM, Papadopoulos P, van Ijcken W (2011). Chronic IFN-γ production in mice induces anemia by reducing erythrocyte life span and inhibiting erythropoiesis through an IRF-1/PU.1 axis. Blood.

[61] Gao R, Chen S, Kobayashi M, Yu H, Zhang Y, Wan Y (2015). Bmi1 promotes erythroid development through regulating ribosome biogenesis. Stem Cells.

[62] Kim J, Hwangbo J, Wong PKY (2011). p38 MAPK-Mediated Bmi-1 down-regulation and defective proliferation in ATM-deficient neural stem cells can be restored by Akt activation. PLoS One.

[63] Vucic D (2013). The role of ubiquitination in TWEAK-stimulated signaling. Front Immunol.

[64] Leng R-X, Pan H-F, Qin W-Z, Wang C, Chen L-L, Tao J-H (2011). TWEAK as a target for therapy in systemic lupus erythematosus. Mol Biol Rep.

[65] Choe J-Y, Kim S-K (2016). Serum TWEAK as a biomarker for disease activity of systemic lupus erythematosus. Inflamm Res.

[66] Sastre C, Fernández-Laso V, Madrigal-Matute J, Muñoz-García B, Moreno JA, Pastor-Vargas C (2014). Genetic deletion or TWEAK blocking antibody administration reduce atherosclerosis and enhance plaque stability in mice. J Cell Mol Med.

[67] Chicheportiche Y, Fossati-Jimack L, Moll S, Ibnou-Zekri N, Izui S (2000). Down-regulated expression of TWEAK mRNA in acute and cchronic inflammatory pathologies. Biochem Biophys Res Commun.

[68] Pettersen I, Baryawno N, Abel F, Bakkelund WH, Zykova SN, Winberg J-O (2013). Expression of TWEAK/Fn14 in neuroblastoma: implications in tumorigenesis. Int J Oncol.

[69] Liebelt BD, Shingu T, Zhou X, Ren J, Shin SA, Hu J (2016). Glioma stem cells: signaling, microenvironment, and therapy. Stem Cells Int.

[70] Dwyer BJ, Jarman EJ, Gogoi-Tiwari J, Ferreira-Gonzalez S, Boulter L, Guest RV (2021). TWEAK/Fn14 signalling promotes cholangiocarcinoma niche formation and progression. J Hepatol.

[71] Hou T, Zhang W, Tong C, Kazobinka G, Huang X, Huang Y (2015). Putative stem cell markers in cervical squamous cell carcinoma are correlated with poor clinical outcome. BMC Cancer.

[72] Zou H, Wang D, Gan X, Jiang L, Chen C, Hu L (2011). Low TWEAK expression is correlated to the progression of squamous cervical carcinoma. Gynecol Oncol.

[73] Di Martino L, Dave M, Menghini P, Xin W, Arseneau KO, Pizarro TT (2016). Protective role for TWEAK/Fn14 in regulating acute intestinal inflammation and colitis-associated tumorigenesis. Cancer Res.

[74] Hayakawa Y, Nakagawa H, Rustgi AK, Que J, Wang TC (2021). Stem cells and origins of cancer in the upper gastrointestinal tract. Cell Stem Cell.

